# Preliminary Identification of Macroconidia (Asexual Forms) of Alternaria Species in Histologic Tissue Sections in a Post Covid Patient with Allergic Fungal Rhinosinusitis

**DOI:** 10.5146/tjpath.2026.13330

**Published:** 2026-01-31

**Authors:** Sateesh Chavan S, Madhuri Dindalkoppa, Purushotham Reddy

**Affiliations:** Department of Pathology, Karnataka Institute of Medical Sciences, Karnataka, India

**Keywords:** Preliminary identification, Alternaria, Macroconidia, Phaeohyphomycosis, Post Covid-19

## Abstract

To document a case of “preliminary” identification of Alternaria sp (a phaeohyphomycotic agent) based on morphology in tissue section in a patient with allergic fungal rhinosinusitis.

A 25-year-old male, a known asthmatic in a post Covid -19 state, presented with headache, facial swelling and nasal block with discharge of brownish mucoid material. Debrided material from the right maxillary antrum and middle turbinate showed brownish mucoid material admixed with firm to hard degenerated bony spicules sent in formalin and subjected for histopathological examination. Histopathology showed fragments of tissue, mucoid material, degenerated bony spicules, and blood clots. Amidst ulcerated epithelium and mucoid debris were seen scattered pigmented fungi in a state of ‘vegetative sporulation’ with characteristic brownish multicellular ‘macroconidia’ diagnostic of Alternaria sp. A diagnosis of “Phaeohyphomycosis” possibly due to Alternaria sp was offered. The patient was treated with Amphotericin B. The patient was lost to follow up.

Clinical materials such as tissue sections or smears from nasal mucus secretions in cases of allergic fungal rhinosinusitis provide a very good source for “preliminary” identification of species and early institution of therapy while waiting for the fungal culture report.

## INTRODUCTION

Asexual forms of molds are called ‘Conidia’ (also ‘spore’ is the generally used term) which are produced by conidiophores. Conidiophores are thick-walled stalks arising perpendicular to hyphae. Conidiophores can bear conidia directly (phaeohyphomycotic agents) or phialides, which in turn give rise to conidia (hyalohyphomycotic agents like Aspergillus sp). Conidiophores are seen in ‘in vitro’ culture or in tissue following vegetative sporulation (mycetoma, phaeohyphomycosis, Rhizopus sp). Conidia can be small single celled (microconidia) or large multicellular (macroconidia) (1,2).

Pigmented molds (dematiaceous fungi) are the most common etiologic agents of allergic fungal rhinosinusitis (AFRS) as opposed to hyalohyphomycotic agents which were once thought as most common agents of AFRS (1–3). Phaeohyphomycotic agents which cause AFRS include *Alternaria* sp, *Bipolaris *sp, *Curvularia* sp, *Drechslera* sp and *Exserohilum* sp. Often they induce production of tenacious allergic mucin, involve superficial epithelium, rarely invade deeply and cause minimal tissue inflammation/tissue response (except for increased mucin production). Identification of these agents is by demonstration of diagnostic ‘macroconidia’ (asexual forms) in clinical material (mucin secretion or histopathology tissue sections) or in ‘in vitro’ fungal culture (2). These macroconidia can be seen in tissue and or mucus secretion when there is ‘vegetative sporulation’.

Here is a case report of ‘preliminary’ identification of *Alternaria* sp in tissue section showing evidence of vegetative sporulation with characteristic ‘macroconidia’.

## CASE REPORT

A 25-year-old male presented with headache and facial swelling for 15 days. On examination, facial swelling was noted on the right nasolabial fold extending over the external nose. Sinus examination showed degenerated brownish mucoid collections in the right middle turbinate, right maxillary antrum and right ethmoid bullae. The opposite side nasal cavity showed mild deviation of nasal septum towards the left side. His routine investigation revealed hemoglobin of 12.7gm/dL, total white blood cell count of 6700/Cumm, and absolute neutrophil count 4.6x109/L; differential leucocyte count showed 62% neutrophils, 24% lymphocytes, 14% eosinophils; platelet count was 113x109/L. The patient was a case of post Covid-19 state one year back. He was a known asthmatic with a history of allergic rhinitis. He underwent debridement of the right maxillary antrum and the middle turbinate which showed brownish mucoid material admixed with firm to hard degenerated bony spicules and it was sent for histopathological examination. Material was not available for fungal culture as it was formalin fixed.

**Figure 1:**
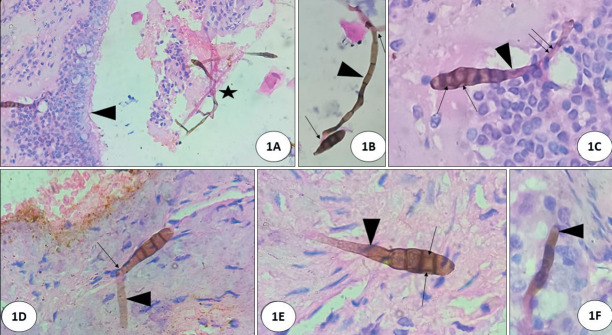
1: Showing fungi in a state of vegetative reproduction (sporulation) with pigmented multicellular macroconidia in surface epithelium & mucus debri (asterix) with minimal inflammation **(A,B)** (x40, H&E). Macroconidia show typical alternating vertical & horizontal septations (black arrows) **(C)** (x200, H&E) with characteristic ‘geniculate’ conidiophore (a kind of ‘knee bend like’ attachment to hyphae) (black arrows & arrow heads) **(D, E)** (x200, H&E); a portion of pigmented septated hyphae seen adjacent to mucous gland **(F)** (x200, H&E) (black arrow head).

**Figure 2:**
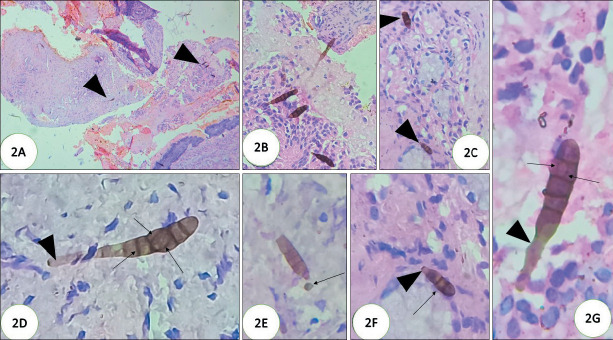
Histopathology of sinus currettings showing numerous released pigmented multicellular macroconidia in surface epithelium & mucus debri (black arrow heads) **(A)** (x10, H&E) **(B)** (inset of A); some adjacent to mucous glands (black arrow heads) **(C,F)** (inset of C)) (x40. H&E); characteristic alternating horizontal and vertical/ oblique septations (black arrows) with their ‘conidiophore’ (black arrow heads) **(D,G)** (x200, H&E); with minimal inflammatory response; occasional macroconidia releasing single celled ‘conidia’ (long black arrow) **(E)** (x200, H&E).

**Figure 3:**
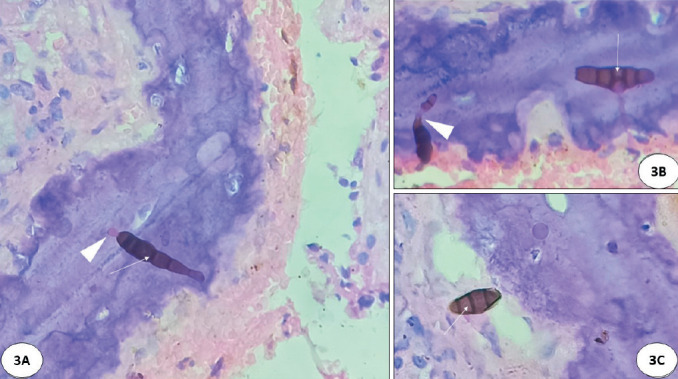
Showing characteristic macroconidia with its conidiophore seen eroding the degenerated nasal osseous tissue with sparse inflammatory response. A macroconidia showing single celled terminal round conidia about to get detached (white arrow head) **(A)** (x200, H&E). Eroding macroconida show typical vertical/oblique and transverse septations alternating each other (hence the name “Alternaria”) (white arrows) resembling ‘hand held grenade’ **(B,C)** (x200, H&E) with their conidiophore (white arrow head) **(B)**.

Histopathology showed fragments of tissue, mucoid material, and blood clots. Tissue fragments showed transitional epithelial lining with ulceration and squamous metaplasia; subepithelial fibrocollagenous tissue showed granulation tissue with congested blood vessels, moderate lymphoplasmacytic infiltration, histiocytes and degenerated bony spicules. Epithelial fragments showed overlying mucoid debris. Amidst ulcerated epithelium and mucoid debris there were scattered pigmented hyphae which were narrow, parallel walled, irregularly septate, and non-branching. Typically, there were foci of ‘sporulation’ (‘vegetative reproduction’) (Figure 1) with characteristic brownish multicellular ‘macroconidia, (asexual forms of fungi) with its attached brown walled ‘stalk’ (conidiophore) (Figure 1). Amidst ulcerated epithelium & mucoid debris there are scattered pigmented hyphae which are narrow, parallel walled irregularly septate and non-branching (Figure 1). The macroconidia with its conidiophore and hyphae were seen in superficial mucosa (Figure 2), some adjacent to mucous glands (Figure 2). They are oval shaped with tapering at one end and rounded at the other end and characteristically showed ‘beaded’ appearance with multiple irregular alternating ‘transverse, oblique and ‘vertical’ septations (Figure 2). Occasional ‘single celled’ ‘microconidia’ were seen detaching from macroconidia (Figure 2). Some were seen eroding underlying osteocartilaginous tissue (Figure 3).

Because of the presence of characteristic macroconidia, a diagnosis of ‘phaeohyphomycosis’ possibly due to *Alternaria* sp was offered. The patient was treated with Amphotericin B for 2 months and was lost to follow up.

## DISCUSSION

Allergic fungal rhinosinusitis is a common condition accounting for about 5-10% of the cases of chronic sinusitis not responding to antibiotics and requiring surgical intervention (3). The most common etiologic agents causing AFRS are *phaeohyphomycotic* agents. Most common phaeohyphomycotic agents causing AFRS and allergic bronchopulmonary mycosis include *Alternaria sp, Bipolaris sp, Curvularia sp, Drechslera asp and Exserohilum *(1–3). Typically, these agents induce excessive thick viscous mucus secretion (allergic mucin often with Charcot laden crystals and eosinophils) with fungal elements including pigmented hyphae and or conidiophore with its characteristic macroconidia (following ‘vegetative sporulation’) (2–5). The present case, an immunocompetent post Covid-19 patient, was a known asthmatic and allergic subject and presented with nasal manifestations with peripheral blood eosinophilia, nasal discharge of thick tenacious viscid mucus, with characteristic macroconidia of *Alternaria *sp both in mucus as well as in mucosa with minimal inflammatory response and with a foci of bone erosion.

*Alternaria *sp are ubiquitous plant pathogens and soil saprophytes. They belong to Phylum *Ascomycota,* Subphylum *Pezizomycotina*, Class *Dothideomycetes* and Order *Pleosporales* (2). Once they were considered nonpathogenic, causing only bronchial asthma in atopic individuals and hypersensitivity reactions. The spectrum of lesions caused by Alternaria include chronic AFRS, allergic asthma, ABPM in immunocompetent hosts, and less often cutaneous and ocular involvement in immunosuppressed hosts (3,5). They frequently colonize macerated/denuded/ or previously injured lesions of skin/mucosa with or without ‘vegetative sporulation’ with release of characteristic macroconidia. These macroconidia are frequently implicated in the allergic reactions (allergic asthma, AFRS, ABPM) and their exacerbations, though their exact contributing role in pathology is doubtful (1). Often, they are restricted to superficial mucosa with or without tissue invasion or with only minimal inflammation (2) or sometimes no inflammation (4). Occasionally, they cause progressive destructive disease particularly in immunocompetent hosts with erosion of underlying osseous tissue with or without a significant inflammatory response or destructive osteomyelitis producing a mass effect (2,4,6,7). Rarely they do cause invasive disease with dissemination, particularly in immunodeficient hosts (4).

**Figure 4:**
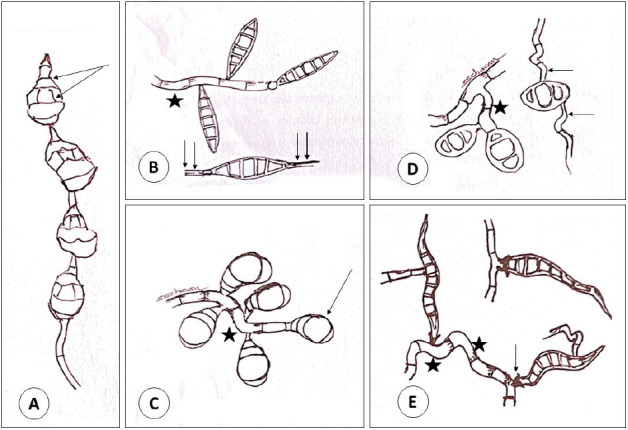
Diagram showing morphology of species-specific ‘diagnostic macroconidia’ of Phaeohyphomycotic agents. **A)** Alternaria sp, typical multicellular macroconidia with alternating vertical/oblique & transverse septations. They are pointed at one end and rounded at the other end, and they grow in chains in culture (sympodial growth). **B)** Bipolaris sp, multicellular ‘fusiform’ macroconidia. They show germination at both ends (double arrows) hence the name. They can have ‘amphigenous’ germination (both from terminal ends as well as from intermediate cells). **C)** Curvularia sp are usually 4 celled macroconidia, typically show swelling (clubbing) of the second cell from the tip creating a ‘curved’ appearance and hence the name. They usually germinate directly from hyphae without hilum or prominent conidiophore. **D)** Drechslera sp, have 3 celled ‘cylindrical’ macroconidia with its narrow ‘conidiophore’, can have amphigenous germination (arrows) and no prominent hilum. **E)** Exserohilum sp have ‘fusiform’ multicellular macroconidia with its conidiophore and ‘ prominent hilum’ (arrow) and hence the name. They can have amphigenous germination. Note the geniculate conidiophore (asterisk).

**Figure 5:**
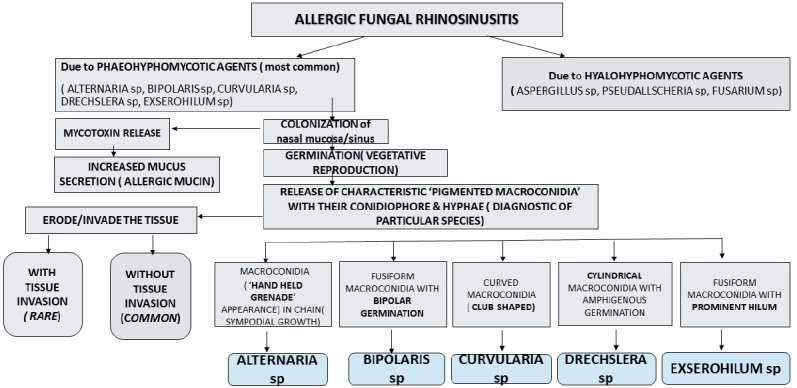
Phaeohyphomycotic agents of allergic fungal rhinosinusitis, their course, and morphological features of species-specific ‘macroconidia’.

Phaeohyphomycotic agents of AFRS reproduce by sporulation whether *in vivo* in ‘open tissue spaces (vegetative sporulation, a phenomenon similar to ‘in vitro’ fungal culture) such as paranasal sinuses, lung cavities which have access to external air, or in ‘closed tissue planes’ (‘adventitious sporulation’) or *in vitro* (by fungal culture) producing characteristic ‘conidia’ (asexual forms) and its conidiophore’, the morphology of which are characteristic for each species and aid in speciation. Detailed morphology of conidia, hilum, origin of germ tubes from basal cells or other conidial cells, location of septa, sequence of conidial septa will be best appreciated in fungal culture (Figure 4) (2–4). Such ‘vegetative sporulation’ in tissue with characteristic conidia and conidiophore help in “preliminary” identification of species of agents before fungal culture. Though ‘adventitious sporulation’ is seen with phaeohyphomycotic agents, it is not commonly observed with *Alternaria* sp. The released macroconidia of phaeohyphomycotic agents of AFRS are multicellular. Their shape range from ellipsoid-fusoid (*Bipolaris, Exserohilum*) to cylindrical (*Drechslera*), curved with transverse septa (*Curvularia*); some grow in chains (sympodial growth) and have alternating horizontal and vertical or oblique septa (*Alternaria*). They germinate from terminal cells (polar germination, ex. *Bipolaris*), both terminal cells and lateral sides of terminal cells (*Exserohilum*) or intermediate cells (amphigenous germination) (*Exserohilum, Drechslera*). The hilum (from which germination occurs) may be prominent and protruding (Exserohilum) or may not be prominent with hila continuous with conidial wall (2). Macroconidia of *Curvularia* are multicellular with 3-4 transverse septations and typically the 2nd cell from the tip undergoes swelling with a thick wall (club shaped), giving a ‘curved’ appearance. Macroconidia of *Alternaria* have multiple alternating horizontal and vertical/oblique septations, and in fungal culture typically grow in chains (sympodial growth; chain of conidia) (Figure 4, Figure 5) (2). Conidiophores that give rise to conidia are usually pigmented, ‘geniculate’ (‘bent knee’ appearance at the site of conidial attachment) and are 4- 9μm wide and up to 30μm long and variably septate (Figure 2) (2).

Radiologically there will usually be multiple sinus involvement with bone erosion in 30-50% of the cases. On Computerized Tomographic scan, usually there is increased attenuation within the sinus, often with serpiginous erosion without tissue invasion (8).

The diagnosis of AFRS is mainly based on collective findings including (1) allergic tenaceous mucin, (2) eosinophils, (3) Charcot-Leyden crystals, (4) pigmented hyphal elements in the mucosa or mucin without significant tissue invasion (3). In the present case we observed the organism in a state of ‘vegetative reproduction’ with asexual forms (macroconidia) with characteristic alternating vertical and horizontal/oblique septations of *Alternaria* species (hence the name) and they looked like ‘hand held grenade’ with its conidiophore (Figure 1, Figure 2, Figure 3). These ‘macroconidia’ were seen often on the surface mucosa without significant inflammation and some were seen eroding the degenerated osseous spicule (2). In potato carrot agar culture, *Alternaria* sp typically grow in ‘chains’ (sympodial growth) (2). In the present case, culture was not possible as the tissue was formalin fixed.

Fungal culture is the ‘gold standard’. These organisms typically produce dark brown velvety colonies on Sabouraud Dextrose Agar showing irregularly septate branching hyphae, which produce yellowish brown conidiophore with terminal or subterminal conidia (vegetative reproduction). Fungal culture may not always be possible because of formalin fixed tissue or the yield may not always be accurate or sometimes not representative of the tissue counterpart due to various reasons including non-viable organisms, homogenization, or previous antibiotic therapy (9).

In tissue or smears from secretions, the organisms of *Alternaria* sp usually show irregularly septate pigmented parallel walled (non-pleomorphic) hyphae with occasional branching in the superficial mucosa without tissue invasion or inflammation, and are difficult to distinguish from other phaeohyphomycotic molds. Occasionally, they are seen eroding the underlying osseous tissue (4,6).

Most of the cases of AFRS due to *Alternaria* sp were managed with surgical curettage alone with removal of tenacious mucus or in combination with azoles such as Itraconazole (2,3).

## CONCLUSION

Clinical materials such as tissue sections or smears from nasal mucus secretions in cases of allergic fungal rhinosinusitis provide a very good source for “preliminary” identification of species and early institution of therapy while waiting for fungal culture results.

## Conflict of Interest

The authors declare that they have no conflict of interest.
